# Effects of the Two-Dimensional Structure of Trust on Patient Adherence to Medication and Non-pharmaceutical Treatment: A Cross-Sectional Study of Rural Patients With Essential Hypertension in China

**DOI:** 10.3389/fpubh.2022.818426

**Published:** 2022-03-04

**Authors:** Yingchao Feng, Shuai Guan, Yanyun Xu, Wenqin Chen, Xianhong Huang, Xiaohe Wang, Meng Zhang

**Affiliations:** Department of Health Management and Policy, School of Public Health, Hangzhou Normal University, Hangzhou, China

**Keywords:** hypertension, Non-pharmaceutical treatment, rural patients, trust, adherence

## Abstract

In rural China, treatment adherence of patients with hypertension remains a challenge. Although early research on patient adherence has confirmed the importance of trust in doctors, the relative contribution and influence of the two-dimensional structure of trust on adherence has not been explored. Thus, this study examined the effects of patient trust in primary care physicians' (PCPs) benevolence and ability on medication adherence, dietary management, and physical activity. The data were derived from 2,533 patients at 54 primary health institutions in China (village level) from February 2017 to May 2018. Participants were assessed using the Chinese version of the Wake Forest Physician Trust Scale and the Therapeutic Adherence Subscale for Hypertensive Patients. Other information included region, gender, age, and self-rated health status. The results of multiple linear regression and structural equation modeling confirmed that patient trust in PCPs' benevolence was positively correlated with patient adherence to medication, diet management, and physical activity. Patient trust in PCPs' ability was negatively correlated with adherence to dietary management and physical activity. We concluded that interventions aimed at increasing PCP benevolence have the greatest potential to improve patient adherence to hypertension treatment. Under the country's policy of advocating to improve PCPs' diagnoses and treatment technology, it may be important to cultivate doctors' communication skills, medical ethics, and other benevolent qualities to improve patients' adherence with drug and Non-drug treatments.

## Introduction

Hypertension is a worldwide public health challenge (1, 2), and by 2025, an estimated two billion people worldwide are expected to suffer from it (1–3). However, epidemiological surveys show that the current global hypertension control rate is only 31.7% (1–3). Meanwhile, China has the largest number of patients with hypertension. Although the government has announced detailed health management service specifications for hypertensive patients, including screening, follow-up visits, classified intervention, and service procedures and requirements, (4, 5), the domestic hypertension control rate is only 16.8% (2). Especially in rural areas of China, hypertension is the chronic disease with the highest prevalence rate (6–8). The prevalence rate exceeds that of urban areas (28.8 vs. 26.9%), but the control rate is much lower than in urban areas (13.1 vs. 19.4%) (2, 8). Thus, improving the blood pressure control rate in China is key to improving the health of its rural populations.

Poor adherence to medication treatment is the main reason for the low rate of hypertension control (3–10), and good medication adherence is the focus of hypertension prevention and control (1, 3). Non-pharmaceutical treatment based on lifestyle changes, including tobacco control, alcohol restriction, moderate physical exercise, and mental stress relief, has also received increasing attention (10–12). A number of experiments show that lifestyle intervention therapies have a clear anti-hypertensive effect (11–13), and a recent study also proved that a healthy lifestyle can reverse the genetic high risk of hypertension in rural people (12). Therefore, it is necessary to evaluate and improve people's adherence to Non-pharmaceutical treatment.

Among the influencing factors of adherence, researchers generally acknowledge the influence of age, education level, occupational status, economic status, trust, and the complexity of treatment methods (8–11, 14, 15). Among these, building trust is the best for optimizing adherence (11, 14). Scholars in various disciplines generally accept that trust is “the willingness of a party to be vulnerable to the actions of another party based on the expectation that the other party will perform a particular action important to the truster, irrespective of the ability to monitor or control the other party” (16, 17). The theory of trust also states that the willingness to trust affects the behavior of participants in an action (15, 18). This helps us understand that patients who trust doctors are more likely to accept their doctor's advice (14, 15, 18). Studies on the relationship between patient trust and patient adherence (8, 13, 19) have expressed the role of patient trust in promoting patient adherence. In other words, trusting doctors plays an active role in both medication and Non-pharmaceutical treatment adherence behaviors of patients.

Notably, many studies have proposed that trust includes at least two dimensions (17, 20–24). For example, McAllister (20) believed that trust can be divided into cognition-based trust and emotion-based trust, and that different types of trust can affect cooperative behavior. Baer et al. (17) highlighted that trust primarily has two bases: trustworthiness and trust propensity, and trustworthiness is the key upstream construct of trust. Barki et al. (24) further demonstrated that the three characteristics of trustworthiness are Non-linearly related to trusting behaviors, and explained the validity and usefulness of the three characteristics of ability, benevolence, and integrity to trust. To measure patients' trust in the field of healthcare, Hall et al. (22) compiled the Wake Forest Physician Trust Scale (WFPTS), including the four dimensions of loyalty, ability, honesty, and kindness. Chinese researchers Dong et al. (23) sinicized the WFPTS based on the psychological and emotional tendencies and actual conditions of Chinese patients, and their reliability and validity tests showed that the scale has a two-dimensional structure of benevolence and ability. Benevolence is the physician 's genuine care for the well-being of the patient (e.g., “My doctor is extremely thorough and careful”) (8, 16, 23). Ability is the physician's capacity to perform the care of the patient competently and reliably (e.g., “My doctor's medical skills are not as good as they should be”) (8, 16, 23). It can be seen that trust encompasses multiple dimensions. A few studies report on the influence of the multi-dimensional structure of trust on patient treatment adherence (25, 26). For example, research by McKee et al. revealed that a physician's benevolence (e.g., respect) and ability to recognize and address the patients' symptoms shaped patient adherence (25). The study by Rozanova et al. (26) shows that benevolence (e.g., positive communication) with clinicians based on mutual respect can improve adherence to antiretroviral therapy for people living with HIV. These studies suggest that patients' trust in a physician's benevolence and ability promotes their adherence to treatment. However, research on the influence of the multi-dimensional structure of trust on patient adherence to medication and Non-pharmaceutical treatment remains scarce, and the relative contributions of different trust dimensions to adherence behavior are inconclusive.

**Figure 1:**
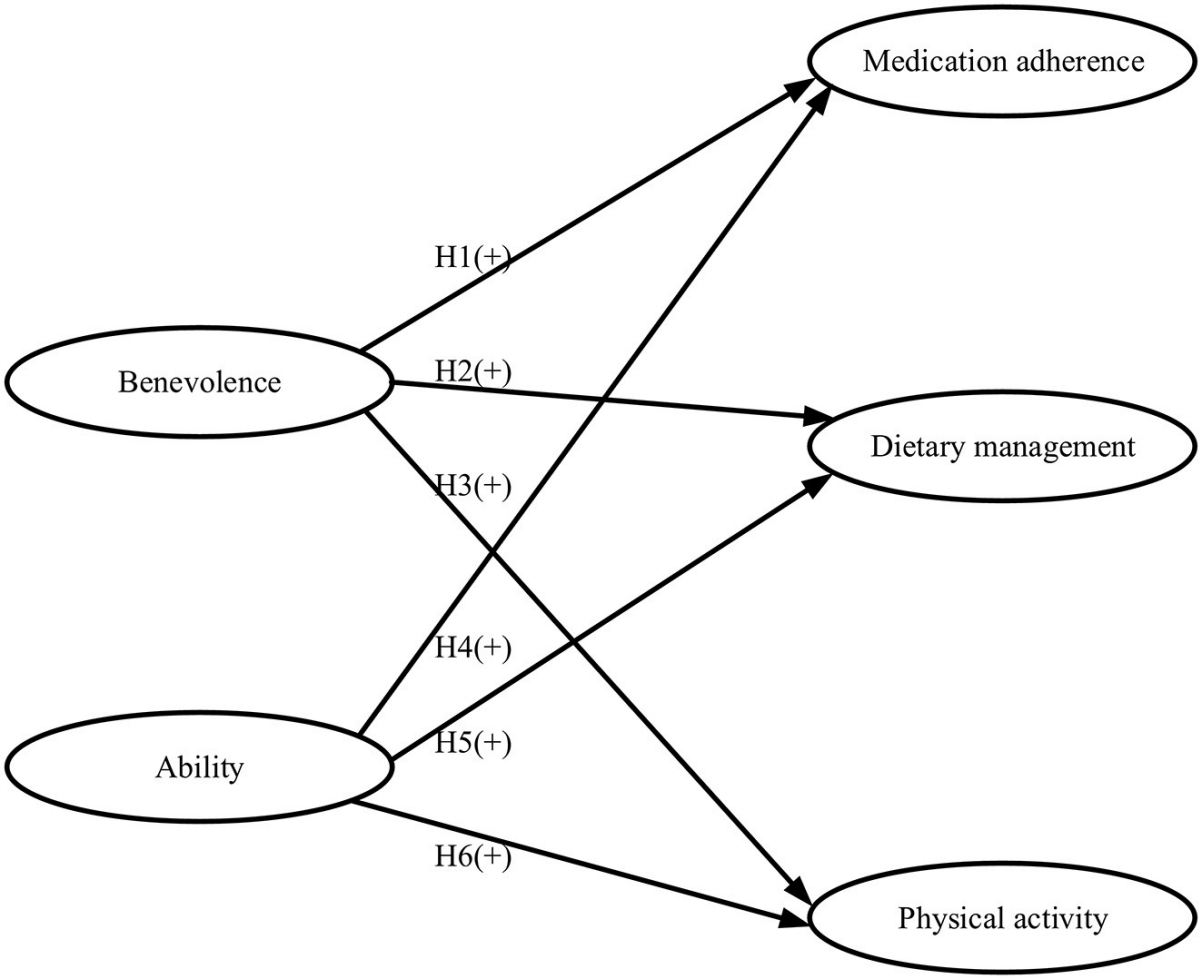
Structural framework of hypothesized relationships.

Therefore, this study explored the effects of trust in primary care physician (PCP) benevolence and ability among patients with hypertension living in China's rural areas on patient adherence to medication and Non-pharmaceutical treatment. For treatment adherence, we investigated patients' medication adherence, dietary management adherence, and physical activity adherence. Regarding patients' trust, we measured how much do patients trust the PCPs' benevolence and ability. As these constructs are designed based on the actual situation of Chinese hypertensive patients, we assumed that the dimensions of patients' trust would have a positive impact on the dimensions of patient adherence to treatment (H1–H6) (The structural framework of the hypothetical relationship is illustrated in Figure 1). The results of this research extend previous reports and provide new insights into the contribution of patients' trust in doctors' benevolence and technology to specific areas of adherence behavior.

## Materials and Methods

### Data Source

Based on geography, we first obtained a representative sample of provinces from eastern, middle, and western China, specifically, Zhejiang, Henan, and Shaanxi, respectively. Next, according to the level of economic development, the counties in each sample province were classified into two categories: high and low. One sample county was randomly selected from each category. Then, in line with the average net income per farmer household, the townships within each sample county were classified into three categories: economically developed, moderately developed, and less developed (27). One sample township was selected at random from each category. Three sample villages were selected within each sample township based on the distance to the township health center (i.e., far, medium, and near). Ultimately, for our survey, 50 patients with hypertension were randomly selected from each sample village using the hypertension management records from the primary health institutions. Random number tables were used to draw samples based on the health file's code. We completed drawing and numbering all random samples prior to the investigation. The rural primary health institutions in China include township health centers, community health service centers, and village clinics/community health service stations (6). Considering the age and education level of the patients with hypertension living in rural areas, the researchers verbally obtained informed consent from the selected patients before conducting the survey. This study was approved by the Research Ethics Committee of AA University.

### Data Collection

Data collection was carried out in two stages using data from patients diagnosed with essential hypertension at rural primary health institutions from February 2017 to May 2018. The diagnostic criteria for essential hypertension were based on the Chinese Guidelines for the Prevention and Treatment of Hypertension (revised version, 2010) (5). The criteria of inclusion were set as follows: (1) patients undergoing treatment for hypertension for more than 1 year; (2) patients with normal IQ; (3) patients without any traumatic brain injury or brain disease, visual or hearing impairment, or mental disorder; and (4) patients who are able to speak and read the Chinese language.

A two-stage investigation was adopted. In the first phase of the survey, 60% of the data collection was completed (28, 29). We used these data to test the reliability and validity of the questionnaire, to complete the revision and design of the scale, to construct a structural framework for the research, and then to propose hypotheses. In the second phase, the remaining 40% of data were collected, and these were used to test the hypotheses. The two-stage investigation used the same measurement tools, while the data came from different samples. The first phase of the investigation was from February to September 2017; 1,547 (response rate: 99.8%; 1,547/1,550) questionnaires were returned; the data were screened by standard deviations of plus or minus 3 (30), and 27 outliers were excluded, which resulted in 1,520 participants. The second phase of the investigation was from October 2017 to May 2018; 1,120 (response rate: 99.6%; 1,120/1,125) questionnaires were returned; seven outliers were excluded, and the final number of participants in the statistical analysis was 1,013. When calculating valid scores for all data, the median was used instead of missing data (8). The groups were comparable in terms of age, gender, and education level.

### Measures

#### Control Variable

The participants were asked questions regarding their sociodemographic characteristics, including residence region, age, gender, marital status, education level, annual per-capita household income, and participation in health insurance. Hypertension health management was assessed with questions regarding the number of follow-up visits made in the past year, blood pressure control, and distance to the nearest health service institute.

#### Independent Variable

Hall et al. (22) verified the reliability and validity of the original WFPTS using a number of extensive empirical studies. The WFPTS contains 10 items that are divided into four dimensions: loyalty (two items), competence (three items), honesty (one item), and benevolence (four items). A modified Chinese version (WFPTS-C) was developed by Dong et al. (23), which includes 10 items divided into two dimensions: benevolence (five items) and ability (five items). The detailed items are presented in Table 1. Items were responded to using a five-point Likert scale ranging from 1 (“strongly disagree”) to 5 (“strongly agree”; e.g., “My doctor will do whatever it takes to provide me all the care I need”; higher scores reflected greater trust). Some items were reverse scored and needed to be adjusted before they were added to the total score (e.g., “Sometimes my doctor does not pay full attention to what I am trying to tell him/her.”). An overall trust score was calculated by adding the unweighted individual item scores; higher scores reflected greater trust. The total scale score ranged from 10 to 50, and the median (quartile) was 24 (4). The Cronbach's α coefficient for the benevolence (ability) factor was 0.789 (0.738).

**Table 1:** Factor analysis with factor loadings for C-WFPTS.

**Factor**	**Items**	**Components**		**Rotation sums of**	
		**(rotation factor loadings)**		**squared loadings**	
		**Factor 1**	**Factor 2**	**% of variance**	**Cumulative %**
**Benevolence**	My doctor will do whatever it takes to provide me all the care I need.	0.720		31.067	31.067
	Sometimes my doctor cares more about what is convenient for him/her than about my medical needs.	0.794			
	My doctor is extremely thorough and careful.	0.714			
	My doctor is totally honest in telling me about all of the different treatment options available for my condition.	0.731			
	My doctor only thinks about what is best for me.	0.667			
**Ability**	My doctor's medical skills are not as good as they should be.		0.737	23.257	54.325
	I completely trust my doctor's decisions about which medical treatments are best for me.		0.517		
	Sometimes my doctor does not pay full attention to what I am trying to tell him/her.		0.752		
	I have no worries about putting my life in my doctor's hands.		0.762		
	All in all, I have complete trust in my doctor.		0.585		

*Kaiser-Meyer-Olkin measure of sampling adequacy: 0.844.*

*Bartlett's test of sphericity: χ2 = 4200.609, p < 0.001*.

#### Dependent Variable

In China, several scales have been developed to evaluate adherence to hypertension treatment. Among these scales, the Therapeutic Adherence Scale for Hypertensive Patients (TASHP), developed by Tang et al. (31), drew on the Adherence of Hypertensive Patients Scale (32) and integrated the definition of adherence—the evaluation of medication adherence of patients with chronic diseases—by Kim et al. (33) and the factors that influence adherence behavior in the case of hypertension to assess adherence to medication and Non-pharmaceutical treatment among patients with hypertension. The TASHP is the first scale that closely fits the Chinese cultural context and that has been used to evaluate treatment adherence among Chinese patients with hypertension.

The TASHP contains 25 items that assess the following four dimensions: poor medication (eight items), medication adherence (five items), lifestyle management (10 items), and tobacco and alcohol management (two items). In this study, the items under the three dimensions of medication adherence, lifestyle management, and tobacco and alcohol management were adopted.

For the 17 items selected from the TASHP, three factors were extracted with eigenvalues >1. Two items with factor loadings below 0.40 were deleted (34, 35), namely, “ensure sufficient sleep” and “regularly monitor blood pressure”. Fifteen items were retained, consisting of the following three dimensions: medication adherence (five items), dietary management (four items), and physical activity (six items). It is consistent with the requirement that in structural equation modeling (SEM), each latent variable must have at least three observed variables to satisfy the identification requirement of the model (34). Among the three dimensions, the dietary management dimension included tobacco and alcohol management behaviors, “less salt or soy sauce in foods, eating less, or no excessively salty food,” and “reducing oil intake and no fatty meat,” based on the results of the principal component analysis. In addition, combining the content of the items and the risk factors influencing health, the other six items were named “physical activity”. The detailed items are presented in Table 2. The Cronbach's α coefficients for the three dimensions ranged from 0.667 to 0.899, with positive correlations among the dimensions that ranged from 0.113 to 0.349 (*p* < 0.01). Since the TASHP was not used directly, we conducted SEM inspection on the abovementioned dimensions. The test results (see Supplementary Table 1) show that the model fits well. Each item was scored on a five-point Likert scale ranging from 1 = “not at all or barely” to 5 = “all the time”. Some items were reverse scored (e.g., “Purchase and take anti-hypertensive drugs on your own according to friend recommendations, advertisements or your own experience”). The total scale score ranged from 15 to 75, with higher scores indicating better adherence. The median (quartile) was 62 (9).

**Table 2:** Factor analysis with factor loadings for TASHP.

**Factor**	**Items**	**Components**			**Rotation sums**	
		**(rotation factor loadings)**			**of squared loadings**	
		**Factor 1**	**Factor 2**	**Factor 3**	**% of variance**	**Cumulative %**
**Medication adherence**	Purchase and take anti-hypertensive drugs on your own according to friend recommendations, advertisements or your own experience.	0.478			24.870	24.870
	Take anti-hypertensive drugs prescribed by your doctor.	0.887				
	Take drugs in the amount as prescribed by your doctor.	0.956				
	Take drugs with the frequency as prescribed by your doctor.	0.957				
	Take drugs at intervals as prescribed by your doctor.	0.916				
**Dietary management**	Pay attention to intake of less salt and soy sauce, no or less salty foods like cured meat products.		0.508		19.558	44.429
	Pay attention to reduction in intake of oil, no fatty foods.		0.506			
	Follow the advice on no or less smoking.		0.865			
	Follow the advice on no or less alcohol.		0.873			
**Physical activity**	Increase the intake of fresh vegetables and fruits.			0.613	12.165	56.593
	Execute regular physical exercise.			0.538		
	Pay attention to weight control.			0.504		
	Try to participate in social activities and interact more with others.			0.655		
	Maintain a positive and optimistic attitude.			0.657		
	Reduce mental stress in various ways, such as watching TV, surfing the Internet, deep breathing, and meditation, etc.			0.752		

*Kaiser-Meyer-Olkin measure of sampling adequacy: 0.790.*

*Bartlett's test of sphericity: χ ^2^ = 13,071.725, p < 0.001*.

### Statistical Analysis

This study adopted a two-step analysis strategy comprising the following steps:

1) Multiple linear regression: Regression analysis was performed with patient adherence to treatment total scale scores as the dependent variables, and the two dimensions of patient trust as independent variables. Additionally, variables associated with the participants' sociodemographic characteristics were used as control variables in this model. Model covariates were selected from those that returned a *p*-value of <0.2 in the univariate analysis.

2) SEM: SEM was constructed to test our hypotheses. Constructing SEM allows for the placement of measurable and latent variables in a generic model. The model can include multiple dependent variables in a single measurement to reduce errors in the multivariate analysis (34). The exogenous observable variables can effectively measure the corresponding latent factors, based on which we could further introduce endogenous factors and their observable variables to construct SEM so that we could quantitatively determine the influence of exogenous latent variables on endogenous latent variables and their importance (34). In this study, benevolence and ability were the exogenous latent variables, and medication adherence, dietary management, and physical activity were the endogenous latent variables in the SEM. Before the analysis, we incorporated the control variable into the regression analysis, standardized it, and replaced the actual adherence scores with standardized predicted values (36). All analyses were performed using SPSS 16.0 and AMOS 22.0 software.

## Results

### Descriptive Analyses

The characteristics of the 1,013 participants are shown in Table 3, which contains complete covariate data. Among them, 31.2% lived in the eastern region, 34.1% in the central region, and 34.7% in the western region. More than half of the participants (63.2%) were women. Most of them were middle-aged or older adults, with a median age of 68 years (41–94 years). The vast majority (79.3%) had an elementary school education or below. However, there is a wide income distribution among them. The largest proportion (46.4%) had hypertension for 4–10 years, while 21.8% had hypertension for 3 years or less. Self-rated health status showed that 42.7% (24.0%) considered their health as good (poor). Most participants had health insurance through the New Rural Cooperative Medical Scheme (78.2%), a medical aid system organized, directed, and supported by the local governments for rural residents (8).

**Table 3:** Characteristics of the participants.

**Characteristic**	***N***	**%**	**Characteristic**	***N***	**%**
**Region**			**Per-capita annual household income**
Eastern province	316	31.2	1 (≤ 1,000 yuan)	214	21.1
Central province	345	34.1	2 (1,001–2,000 yuan)	209	20.6
Western province	352	34.7	3 (2,001–3,916 yuan)	191	18.9
**Gender**			4 (3,917–10,000 yuan)	243	24.0
Male	373	36.8	5 (>10,000 yuan)	156	15.4
Female	640	63.2	**Distance to nearest health institute**
**Age**			<1 km	789	77.9
<45	8	0.8	1–2.99 km	210	20.7
45–59	189	18.7	≥3 km	14	1.4
60–74	585	57.7	**Course of disease**		
≥75	231	22.8	≤ 3 years	221	21.8
**Marital status**			4–10 years	470	46.4
Married	816	80.6	11–20 years	269	26.6
Other	197	19.4	>20 years	53	5.2
**Education level**			**Self-reported health status**		
Primary or lower	804	79.3	Bad	243	24.0
Junior high school	175	17.3	Fair	337	33.3
Senior high school or above	34	3.4	Good	433	42.7
**Medical insurance type**			**Blood pressure control**		
For urban employees	16	1.6	Controlled	652	64.4
For urban residents	67	6.6	Uncontrolled	361	35.6
Basic medical insurance for urban and rural residents	129	12.7	**No. of visits in past year**		
New rural cooperative medical scheme	792	78.2	<4	471	46.5
Other	9	0.9	≥4	542	53.5

### Multiple Linear Regression

Table 4 shows the results of the multiple linear regression analysis predicting patient adherence to treatment. After controlling for other covariates, it was found that the trust score (β = 0.076, *p* = 0.016) was correlated with a significantly higher treatment adherence score. We performed a second linear regression and used the two dimensions of trust as independent variables; this analysis showed that the score of “benevolence” was correlated with a significantly increased treatment adherence (β = 0.208, *p* < 0.001), while “ability” was associated with significantly reduced treatment adherence (β = −0.108, *p* = 0.001). We also found that those who lived in the central province (β = −0.096, *p* = 0.012) reported lower adherence scores. Patients with self-reported health status as fair (β = −0.095, *p* = 0.006) or bad (β = −0.071, *p* = 0.037) had lower adherence. Female patients were more likely to give a higher score for adherence (β = 0.132, *p* < 0.001).

**Table 4:** Results of linear regression models examining predictors of hypertensive patients' treatment adherence with PCPs.

**Variable**	**Model 1**			**Model 2**		
	**(independent variable: total trust score)**			**(independent variable: two dimensions of trust)**		
	**Standardized beta (β)**	**95% Confidence interval**	***P***	**Standardized beta(β)**	**95% Confidence interval**	***P***
**Constant**	—	(46.593,59.566)	<0.001	—	(42.970,55.944)	<0.001
Trust	0.076	(0.030,0.293)	0.016	—	—	—
Benevolence	—	—	—	0.208	(0.485,0.927)	<0.001
Ability	—	—	—	−0.108	(−0.588, −0.153)	0.001
**Region**						
Eastern province (Ref)						
Central province	−0.096	(−2.581, −0.332)	0.012	−0.089	(−2.455, −0.243)	0.018
Western province	−0.032	(−1.597,0.630)	0.503	0.007	(−1.012,1.211)	0.708
**Age**						
≥75 (Ref)						
<45	−0.021	(−6.687,3.330)	0.525	−0.011	(−5.827,4.034)	0.739
45–59	0.077	(−0.014,2.834)	0.063	0.087	(0.191,2.993)	0.053
60–74	0.035	(−0.610,1.612)	0.441	0.038	(−0.538,1.646)	0.384
**Course of disease**						
>20 years (Ref)						
≤ 3 years	−0.088	(−3.684,0.636)	0.158	−0.090	(−3.681.0.566)	0.141
4–10 years	−0.120	(−3.761,0.314)	0.094	−0.118	(−3.704,0.301)	0.092
11–20 years	−0.046	(−2.839,1.350)	0.483	−0.043	(−2.756,1.362)	0.503
**Self-reported health status**						
Good (Ref)						
Fair	−0.095	(−2.460, −0.431)	0.006	−0.090	(−2.370, −0.375)	0.008
Bad	−0.071	(−2.313, −0.067)	0.037	−0.075	(−2.360, −0.151)	0.025
**Gender**	0.132	(1.052, 2.872)	<0.001	0.130	(1.037,2.826)	<0.001
**No. visits in past year**	0.047	(−0.216,1.568)	0.112	0.030	(−0.453,1.309)	0.284
**R** ^**2**^	0.052		0.085	

*Ref, reference group*.

### Structural Equation Model

**Figure 2:**
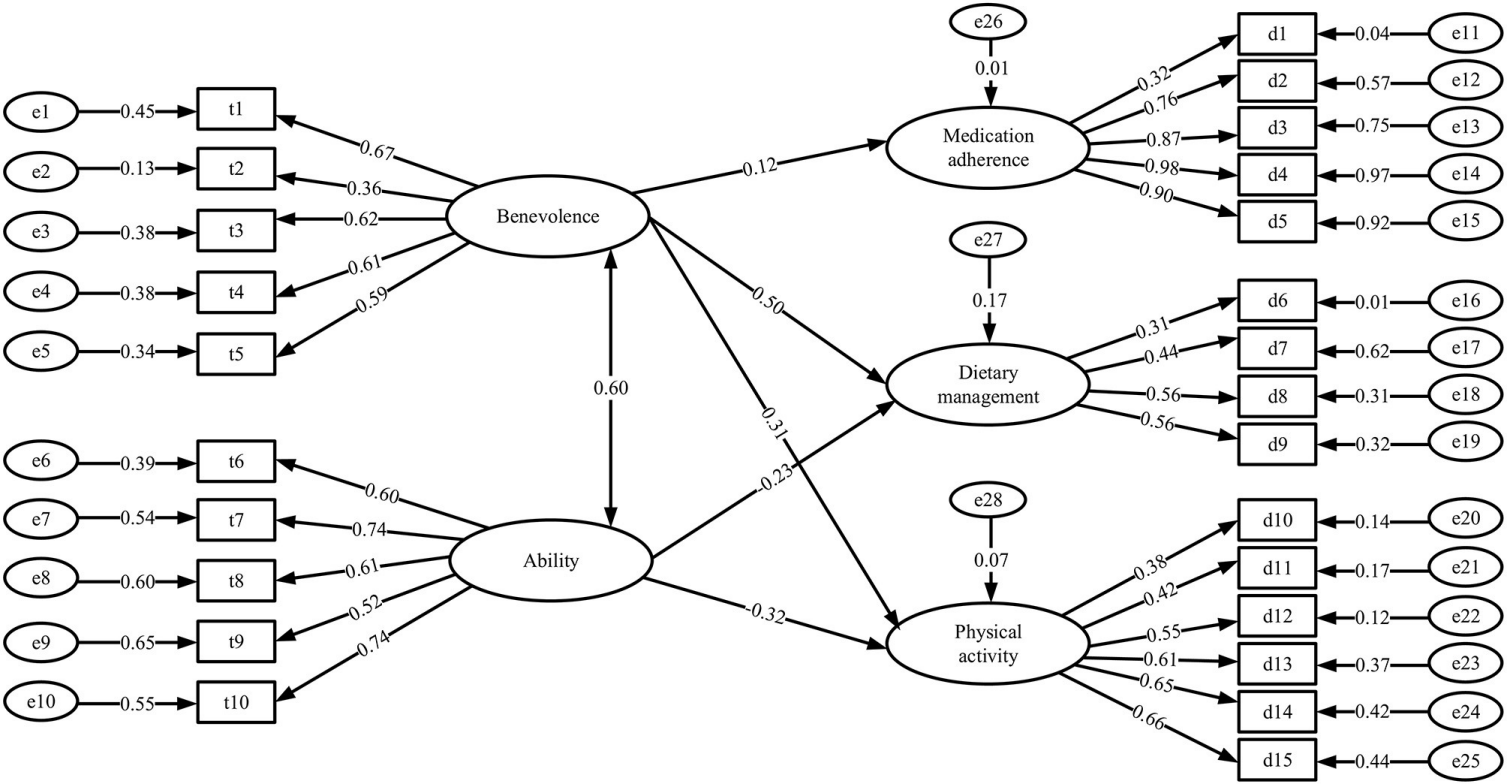
Structural equation model of trust in physicians and patient treatment adherence.

The SEM is illustrated in Figure 2. There was no statistically significant effect of patients' trust in the ability of PCPs on patient medication adherence. Therefore, this pathway was not presented in the model. Table 5 presents the results of the fitting indices. The goodness of fit index (GFI), adjusted goodness of fit index (AGFI), comparative fit index (CFI), and incremental fit index (IFI) values were all above 0.9, and the root mean square error of approximation (RMSEA) value was 0.039. The normed fit index (NFI) was 0.879, which was lower than the recommended standard of 0.9 but within an acceptable range (34, 36). In general, the SEM constructed in this research fit the data well. As shown in Table 6, patients' trust in PCPs' benevolence had a positive and direct impact on their medication adherence (0.118), adherence to dietary management (0.498), and adherence to physical activity (0.314). Patient trust in PCPs' ability had a negative direct impact on patients' adherence to dietary management (−0.231) and physical activity (−0.320).

**Table 5:** Results for SEM fit.

**Fit indices**	**Reference value**	**Model value**
χ2/df	<5.00	2.93
GFI	>0.90	0.93
AGFI	>0.90	0.92
CFI	>0.90	0.91
NFI	>0.90	0.88
IFI	>0.90	0.91
RMSEA	<0.05	0.04
SRMR	<0.05	0.05

*χ2/df, a chi-squared freedom ratio; GFI, goodness of fit index; AGFI, adjusted goodness of fit index; CFI, comparative fit index; NFI, normed fit index; IFI, incremental fit index; RMSEA, root mean square error of approximation; SRMR, standardized root mean square residual*.

**Table 6:** Results of structural equation modeling.

**Path**	**Unstandardized regression weights**	**Standardized** **regression weights**	***t***	**Hypothesis supported**
Benevolence → Medication adherence	0.065	0.118	2.111^*^	Yes
Benevolence → Dietary management	1.007	0.498	6.386^*^	Yes
Benevolence → Physical activity	0.451	0.314	4.449^**^	Yes
Ability → Medication adherence	−0.030	−0.060	−1.118	No
Ability → Dietary management	−0.294	−0.231	−3.446^*^	No
Ability → Physical activity	−0.572	−0.320	−4.194^**^	No

* *p < 0.05.*

** *p < 0.01*.

## Discussion

In this study, we used SEM to clarify the impact of hypertensive patients' trust in PCPs' benevolence and technology on adherence to medication, dietary management, and physical activity. The results show that the level of trust in PCPs' benevolence can predict the above three adherence behaviors of patients to a certain extent. Trust in PCPs' technical level had a certain negative impact on patients' dietary management and physical activity adherence behavior.

When examining the influence of sociodemographic and other variables, we found that patients living in the eastern provinces of China have higher treatment adherence. On the one hand, this regional difference may be caused by different medical information conditions and education levels of residents (2, 8). For example, patients in eastern provinces may have better educational backgrounds and more channels to gain hypertension-related knowledge, which may lead to increased demand for medical participation and increased interaction with PCPs, thereby resulting in higher treatment adherence by patients. On the other hand, these results may be attributed to differences in the population and the number of hypertensive patients in the provinces from which the data were sourced. The total population of Henan Province is higher than that of Zhejiang Province and Shaanxi Province (37), but the number of medical care per 1,000 people is lower than that of Zhejiang Province and Shaanxi Province (38). In addition, a recent survey on the status of hypertension in China (39) showed that the prevalence of hypertension in Henan Province (24.5%) was higher than that of Shaanxi Province (23.9%) and also higher than that of Zhejiang Province (21.9%). We speculated that primary care physician in Henan Province may not have enough energy and time to communicate sincerely with patients and cannot consider the emotional needs of patients. Therefore, patients with hypertension living in the central region in this study had lower adherence. Regarding gender, our research shows that women have higher adherence to their PCPs' instructions. This is consistent with most research findings (7, 8, 19, 40) and may be because women's body resistance is relatively weak, such that they are keener on practicing self-care. Patients with good self-rated health have higher treatment adherence and tend to be more energetic and motivated to maintain this status quo (12, 41).

The results show that with region, gender, age, and self-rated health status controlled for, patient trust has a significant positive impact on patient treatment adherence. Trust in PCP benevolence is the strongest predictor of patient treatment adherence. A high level of trust in PCP benevolence denotes affirmation of the PCP's moral qualities such as careful consideration, patient rights protection, sincere communication, and good attitude. These findings also confirm the conclusions of previous studies that many patient complaints have nothing to do with the doctor's skills. “Softer” qualities such as care, communication, and empathy are important to patients, and they affect patients' adherence to doctors' instructions (8, 11, 14, 15, 18). We also found that patients who have greater trust in PCP benevolence have higher adherence to medication, dietary management, and physical activity. Similarly, Greviskes et al. (42) found that doctors' benevolence can effectively encourage patients with Parkinson's disease to persist in exercising. Matpady et al. (43) considered it desirable for physicians to be aware of emotional factors when advising people with type 2 diabetes mellitus regarding dietary management. Therefore, primary care physician involved in hypertension management should exercise benevolence toward their patients.

Regarding patients' trust in PCPs' ability, our multiple linear regression analysis and SEM results consistently show that “ability” has a small negative impact on patients' treatment adherence. This is inconsistent with the research results of Fung et al. (44), whose survey of 304 adult consumers living in Los Angeles County showed that when PCPs with different technical and interpersonal qualities were simultaneously available, more people chose to follow the advice of PCPs with higher technical qualities. This difference may be attributed to the research object. Our research subjects were mostly older patients from rural China, and because of educational, social, and environmental factors, their self-care awareness was poor, which may have led to excessive dependence on doctors, i.e., they believe that the treatment plan chosen by their physician is best for them and that the physician is competent enough to resolve their disease (8, 23, 40, 45). As such, these patients were not too concerned about the consequences of Non-adherence behaviors (23, 40, 45), which in turn may lead them to engage in more Non-adherence behaviors. This speculation is supported by a study by Lu et al. (41), who found that the probability of treatment adherence increased by five times for patients with greater concerns about health consequences. Overall, while current research on the service quality of primary medical institutions in China aims to improve the diagnosis and treatment ability of PCPs, our research results suggest that we should also focus on the benevolence of physicians (e.g., improve communication and attitude), which is directly perceived by patients.

However, in the analysis of the three dimensions of adherence, the SEM did not show an effect of patients' trust in the ability level of PCPs on the patients' medication adherence. This is probably because China has established strict standards for the treatment of patients with essential hypertension (4, 5). If primary care physicians strictly follow the norms and guidelines to diagnose and treat patients, the differences in physician ability may not be obvious to patients. It is worth considering that hypertension is a disease that requires long-term monitoring and self-management, and rural patients with hypertension tend to be doubtful and dependent, procrastinate during the course of treatment, have poor awareness of health and of the consequences of the disease, and lack attention to their illness. Therefore, it is important to help patients understand that they must play a leading role in their own treatment (46, 47). At present, widely used models of hypertension management worldwide emphasize joint efforts by the government, society, and individuals.

Several limitations should be considered when interpreting the findings of this study. This was a cross-sectional study; therefore, causality could not be determined. In addition, the fit index (R^2^) of the regression model was relatively small, which indicates a limited ability to explain changes in the dependent variable. Treatment adherence among patients with hypertension is affected by a variety of personal, social, physiological, and psychological factors. The interpretation of the independent variables in this study does not exhaust all possibilities and thus requires further evaluation. Despite these shortcomings, we have extended the current literature concerning relationships between patient trust and treatment adherence among patients with hypertension in rural China, thereby clarifying the influence mechanism of the internal dimension.

## Conclusions

This study supports the finding of existing literature that patient trust has a positive relationship with patient adherence. Its main contribution is that it expands the knowledge on Non-pharmaceutical treatment adherence of patients with essential hypertension in rural China, and illustrates the influence of the two-dimensional structure of trust on the three-dimensional structure of treatment adherence. Patients' trust in PCP benevolence has a positive impact on their adherence to medication, diet management, and physical activity, while patients' trust in PCP ability has a minor negative impact on patients' adherence to diet management and physical activity. Therefore, PCPs should pay attention to their “soft” qualities such as a good attitude and communication skills. In addition, self-reported health status has auxiliary significance in predicting the patient's adherence to treatment.

## Data Availability Statement

The raw data supporting the conclusions of this article will be made available by the authors, without undue reservation.

## Ethics Statement

The studies involving human participants were reviewed and approved by Research Ethics Committee of Hangzhou Normal University. Written informed consent for participation was not required for this study in accordance with the national legislation and the institutional requirements.

## Author Contributions

YF and MZ: conceptualization. YF, MZ, and WC: methodology. YF and XH: software. SG, WC, and XH: validation. YF and YX: formal analysis. YF, SG, YX, WC, and MZ: investigation. YF, SG, and YX: writing original draft preparation. MZ, XH, and XW: writing—review and editing. XW: supervision. MZ and XW: funding acquisition. All authors have read and agreed to the published version of the manuscript.

## Funding

This study was supported by the National Natural Science Foundation of China (Grant Nos. 71403075 and71974050).

## Conflict of Interest

The authors declare that the research was conducted in the absence of any commercial or financial relationships that could be construed as a potential conflict of interest.

## Publisher's Note

All claims expressed in this article are solely those of the authors and do not necessarily represent those of their affiliated organizations, or those of the publisher, the editors and the reviewers. Any product that may be evaluated in this article, or claim that may be made by its manufacturer, is not guaranteed or endorsed by the publisher.
