# Supplementary material for: Effects of the Two-Dimensional Structure of Trust on Patient Adherence to Medication and Non-pharmaceutical Treatment: A Cross-Sectional Study of Rural Patients With Essential Hypertension in China
Source: Front Public Health. 2022 Mar 4;10:818426. doi: 10.3389/fpubh.2022.818426 (PMC8931731; doi:10.3389/fpubh.2022.818426)
Supplement: Supplementary file 1 [file Table_1.docx]

Supplementary Material

**Supplementary Table 1 |** Fit indices of patient treatment adherence model.

| Fit indices | Reference value | Model value |
| --- | --- | --- |
| χ2/df | ＜5.00 | 4.79 |
| GFI | ＞0.90 | 0.97 |
| AGFI | ＞0.90 | 0.95 |
| CFI | ＞0.90 | 0.98 |
| NFI | ＞0.90 | 0.97 |
| IFI | ＞0.90 | 0.98 |
| RMSEA | ＜0.05 | 0.05 |
| SRMR | ＜0.05 | 0.05 |

χ2/df, a chi-squared freedom ratio; GFI, Goodness of fit index; AGFI, adjusted goodness of fit index; CFI, comparative fit index; NFI, normed fit index; IFI, incremental fit index; RMSEA, root mean square error of approximation; SRMR, standardized root mean square residual.
